# Assessing the Prognostic Value of Gait Speed to Functional Milestones Status Post Total Hip and Knee Arthroplasty

**DOI:** 10.7759/cureus.96321

**Published:** 2025-11-07

**Authors:** Ryan C McCoy, Matthew M Crowe, Andreas Remis, Anthony Paz, Ian Luther

**Affiliations:** 1 Physical Therapy, Mayo Clinic, Jacksonville, USA; 2 Orthopedics, Mayo Clinic, Jacksonville, USA; 3 Physical Therapy, Duke University, Durham, USA

**Keywords:** gait speed, outcome measure, physical medicine and rehabilitation, physical therapy rehabilitation, total joint replacement

## Abstract

Introduction: Summative patient outcome education is consistently presented to patients following total joint replacement on set and distant timelines away from the date of surgery to better manage their expectations. However, these distant timelines may not accurately reflect the individuals who can regain functional abilities at earlier time frames. It would be insightful to explore if gait speed assessment, following total joint replacement, correlates with the specified timelines patients need to achieve functional milestones status post total hip and knee arthroplasty. The primary aim of this study is to explore if a postoperative three-meter walk test provides prognostic value for the time participants take to achieve functional milestones status post total hip or knee replacement.

Methods: This study was an observational cohort study with target accrual set to 100 participants. Inclusion criteria were patients who underwent total hip or knee arthroplasty and attended inpatient and outpatient physical therapy at a surgical institution. Exclusion criteria were patients who lacked the ability to provide informed consent, patients with postoperative complications following total joint replacement, revision surgeries, or non-compliance with scheduled physical therapy. Gait speed was assessed within one to three days following total hip or knee replacement and participants were provided a survey where they recorded the date of when they achieved the specified milestones. Continuous variables were summarized as median (range) while categorical variables were reported as frequency (percentage). Patients were divided into two groups based on their gait speed with the median value of gait speed as the cutoff point. Wilcoxon rank sum test was used to compare continuous variables between groups with higher and lower gait speed and Fisher’s exact test was used to compare the categorical variables. All tests were two-sided with a p value of <0.05 considered statistically significant.

Results: No statistically significant correlations were found between gait speed and days to achieving functional milestones in this sample.

Conclusion: There were multiple limitations contributing to the results of this study. Poor retention rate, which led to a small sample size limiting the power of the study, was the primary limitation. Further exploration is warranted to better understand if gait speed assessment provides prognostic value for functional milestones following total hip or knee replacement.

## Introduction

Patient inquires following a total joint replacement commonly include questions revolving around expected pain experiences, functional capabilities, and recovery timelines. Preoperative pain and function are the greatest predictors of pain and function at six months following lower extremity arthroplasty [1]. Recent systematic reviews have corroborated these findings for total knee and hip arthroplasties [2,3]. These individuals with higher preoperative pain and lower perceived function generally experience greater relative improvements in pain, function, and satisfaction compared to those with lower levels of preoperative pain and functional impairment [1,4,5]. Many studies provide valuable insight into specified outcomes following total knee and hip arthroplasties at set timelines away from the date of surgery. Postoperative recovery counseling is typically provided at set and distant timelines. Current research regarding patient satisfaction and overall recovery status post total joint replacement aligns with this education strategy to better manage expectations [1-5]. At times, however, these distant timelines may not sufficiently satisfy the patient's inquiry, nor accurately reflect the individuals who can regain functional abilities at earlier time frames. From this lens, it would be insightful to explore if a standardized functional performance measure during initial physical therapy assessment can provide predictive insight into the achievement of common functional milestones status post total hip and knee arthroplasty.

Our literature review revealed several studies that examined the prognostic value of postoperative measures on outcomes. One study examined the relationship between postoperative day two Timed Up and Go (TUG) scores with length of stay and functional status at two weeks following total knee or hip arthroplasty [6]. They found postoperative day two TUG cut-off scores of 31.5 and 30.9 seconds predicted length of stay and functional status at week two, respectively. However, limitations included that they did not explore other factors associated with function following hospital discharge and the prognostic indicator was limited to two weeks postoperatively. Another study of total knee arthroplasties examined the predictive value of postoperative day two knee range of motion and TUG scores on six-month motion and six-minute walk test (6MWT) outcomes [7]. Postoperative day two TUG scores provided prognostic value for 6MWT performance at six months, but day-two knee motion did not provide prognostic value for six-month motion. The authors speculated that day-two knee range of motion may not provide great prognostic value due to patient differences in pain tolerance, anesthesia type, and postoperative pain medication dosing. In contrast, other studies show postoperative motion measured on days five through eight does predict long-term motion [8-10]. Interestingly, Bade et al. found that preoperative knee range of motion and function were of greater utility in long-term prognosis than postoperative day-two knee range of motion and function [7]. Additionally, a recent systematic review showed that preoperative measurements such as knee range of motion have uncertain impacts on postoperative clinical outcomes [4]. Practically speaking, preoperative values are also not often available to clinicians postoperatively particularly for the majority of outpatient physical therapy clinics. Therefore, postoperative measures may be most pragmatic to base prognoses on for those respective healthcare providers.

With consideration of post operative prognostic measures, research shows greater postoperative total hip arthroplasty function is associated with greater postoperative hip range of motion and that greater postoperative total knee arthroplasty function is associated with greater postoperative improvement in range of motion, strength, ambulation distance, and sit to stand testing [1,4,5]. Normative gait speed values are well established in the literature [11]. One meta-analysis highlights that gait speed is an important predictor for varying outcomes across differing populations [11]. Further review into the prognostic value of gait speed for the postoperative total joint populations reveals a scarcity of literature. One study utilized functional performance testing to help identify functional trajectories following a total joint knee replacement [12]. In this study, gait speed was not assessed, and they took into account multiple variables which were measured repeatedly at set times utilizing a latent class mixture model for analysis [12]. One interesting study was found that explored gait speed as a predictor for function following total knee replacement, but the study design correlated gait speed classifications to predicting function based solely on TUG testing results [13]. This study summarized that clinical relevance of classifying patients based on walking speed remains unclear, as it did not predict short- and long-term functional outcomes [13]. Despite these reviewed findings, it is evident there is a need for further exploration into the prognostic value of gait speed performance to specified functional milestones following a lower extremity total joint replacement. Addressing this gap in the literature could have a meaningful impact for healthcare system management and at the individual level for healthcare providers to provide tailored patient counseling on unique recovery timelines following total knee or hip replacement.

The three-meter walk test (3MWT) is a functional performance measure that is commonly utilized in physical therapy for assessing gait speed. Physical therapy functional performance testing varies between institutions, but short-distance gait speed assessment is a simple test to administer during standard physical therapy assessment on a postoperative population. It would be valuable to explore whether the 3MWT correlates with timelines for a patient to achieve specified functional milestones following a total joint replacement. Intuitively, the authors speculate that patients who initially ambulate at a self-selected quicker pace may be an indication for quicker recovery and may correlate to the achievement of functional milestones at a more rapid pace. The primary aim of this study was to explore if the 3MWT, assessed following a total joint replacement, provides prognostic value into the time participants take to wean from a walker, wean from all assistive devices, navigate stairs reciprocally, walk in community for 30 minutes or one mile, and lastly the time taken for the patient to wean from pain medication.

## Materials and methods

This study was a non-funded observation cohort study with a target accrual of 100 subjects. This study attained Institutional Review Board approval at Mayo Clinic Florida (IRB 18-009437) April 19, 2019. Consent for participation was attained by the primary author for all participants in this study. The subject population was a convenience sample of patients referred by the surgical institution to the affiliated outpatient physical therapy setting following total hip or knee arthroplasty. Inclusion criteria were patients who underwent total hip or knee arthroplasty without complication, completed inpatient physical therapy, participated in outpatient physical therapy at affiliated hospital within three days from hospital discharge, and were able to ambulate during their initial physical therapy consultation. Exclusion criteria were patients who lacked the ability to provide informed consent, participants deemed to have acute postoperative complications not considered to be normal recovery following a total joint replacement, or patients who underwent revision surgery. 

Gait speed is the time an individual takes to walk a specified distance on a level surface, with or without an assistive device, over a short distance. The 3MWT involves timing the individual's self-selected walking pace in meters per second over a total distance of three meters. Ideal testing parameters allow additional space for at least two meters for acceleration and two meters for deceleration to standardize the assessment and improve the reliability of the test. Participant gait speed was calculated when the first foot crossed the beginning and ending point of the set distance. Each participant walked this specified distance twice in an open hallway to allow for accommodation to walking in that environment with the latter being recorded. The 3MWT was assessed during the participants' inpatient and outpatient physical therapy visits. The recorded gait speed was taken from the outpatient physical therapy initial consultation, which was within the first three days from date of surgery. Participants were instructed to walk at a self-selected pace with the physical therapist standing posterolateral to the patient for standby assist down a 60-foot hallway. The physical therapist recorded the individual's gait speed across 3 meters, which was marked at the middle of the hall. Standard physical therapy evaluation was completed in addition to gait speed assessment across all participants following their total hip or knee replacement. Upon finishing the outpatient physical therapy consultation, participants were informed of the study to reduce bias of efforts during gait speed testing. Consented participants were then provided a printed functional log, or survey. This survey listed five milestones with instructions for the participant to fill in the date they were able to accomplish each specified milestone. Participants were tasked with recording the dates for each achieved milestone and returning the survey upon completion to the primary investigator via email or fax, which was labeled on the printed survey. The milestones included: when the participant weaned from their walker, when the participant weaned from all assistive devices, when the participant was able to reciprocally navigate stairs, when the participant was able to walk in the community one mile or 30 minutes, and when the participant weaned from all pain medication.

Continuous variables were summarized as median (range) while categorical variables were reported as frequency (percentage). Patients were divided into two groups based on their gait speed with median value of gait speed as the cutoff point. Wilcoxon rank sum test was used to compare continuous variables between groups with higher and lower gait speed and Fisher’s exact test was used to compare the categorical variables. All tests were two-sided with p value of <0.05 considered statistically significant.

## Results

This study explored whether gait speed, assessed with the 3MWT during outpatient physical therapy examination, had prognostic value for predicting an individual's ability to achieve specified functional milestones following a total hip or knee replacement. No statistically significant correlations were found between gait speed and days to functional milestones in this sample. Of the consented 100 participants, 26 participants returned the functional survey log. Enrolled patients included a diverse group of male and female participants at least 37 years of age or older regionally located throughout the Southeast. Ages of participants ranged from 36-70 years of age with 15 male participants and 11 female participants. From this sample, 38.5% of participants had a total knee replacement and 61.5% had a total hip arthroplasty. Table 1 depicts this demographic summary with continuous variables (participants' age and gait speed) summarized as median with respective ranges and the categorical variables (gender and joint replacement) shown as percentages. In Table 2, participants were divided into two groups based on their gait speed over or under with the median value of 0.43 m/s as the cutoff point. Time to achieve each milestone was reflected for both groups and shown by the median days each group took to achieve the specified milestone with respective ranges. Wilcoxon rank sum test was used to compare continuous variables between the groups and Fisher’s exact test was used to compare the categorical variables. 

**Table 1:** Demographic Summary N=number of participants

Age	Overall (N=26)
N	26
Median (Range)	61.0 (36.0, 70.0)
Gender	
N	26
Male	15 (57.7%)
Female	11 (42.3%)
Joint	
N	26
Knee replacement	10 (38.5%)
Hip replacement	16 (61.5%)
Gait speed in meters per second	
N	26
Median (Range)	0.43 (0.09, 1.03)

**Table 2:** Days to Functional Milestones by Gait Speed Classification N=number of participants AD=assistive devices min=minutes

	Gait speed >= 0.43 (N=13)	Gait speed < 0.43 (N=13)	Total (N=26)	P value
Days off medications				0.64
N	13	13	26	
Median (Range)	13.0 (2.0, 74.0)	16.0 (1.0, 55.0)	15.0 (1.0, 74.0)	
Days off walker				0.33
N	13	13	26	
Median (Range)	11.0 (3.0, 22.0)	12.0 (6.0, 32.0)	12.0 (3.0, 32.0)	
Days off all AD				0.61
N	13	13	26	
Median (Range)	21.0 (3.0, 38.0)	16.0 (8.0, 39.0)	20.0 (3.0, 39.0)	
Days using stairs				0.23
N	13	13	26	
Median (Range)	26.0 (3.0, 44.0)	17.0 (7.0, 32.0)	21.5 (3.0, 44.0)	
Days walking mile/30min				0.57
N	13	13	26	
Median (Range)	32.0 (5.0, 58.0)	31.0 (10.0, 87.0)	31.0 (5.0, 87.0)	

## Discussion

Statistical analysis showed no significant findings when comparing gait speed groups to specified milestones and therefore inferences from this study should not be made to the total hip or knee patient population. The greatest limitation contributing to the results of this study was a low sample size due to poor survey return rate. The sample group included a diverse group of male and female participants regionally located throughout the Southeast United States who sought a surgical medical destination site. In addition to the low sample size, analysis included patients with hip or knee replacements which further limits inferences for each group. From the 100 consented participants, only 26 returned the functional survey log. Additional steps could have been taken to improve the 26% survey return rate. Reducing the burden for participants to return surveys via email or fax, consideration of investigator-generated survey submission reminders, consideration of participant remuneration, or more frequent provider follow-ups at set timelines could improve retention rate.

Our results show great variability in both groups for the days taken for participants to wean from pain medications. The faster gait speed group weaned from pain medication with a median value of 13 days while the slower gait speed group weaned from pain medications with a median value of 16 days. Range of days for each group was large with the faster gait speed group weaning from pain medications as early as day two up to day 74 and the slower gait speed group weaning as quickly as day one up to day 55. Median days taken to wean off the walker and walking one mile or 30 minutes were nearly identical between groups, though ranges varied between groups. Median days to wean from all assistive devices was 21 days and 16 days for the faster and slower gait speed group, respectively, which were similar ranges that contrasted our initial hypothesis. Median days to use stairs reciprocally was 26 days for the faster speed group in comparison to 17 days for the slower gait speed group, with noted variation in ranges between groups. No inferences or predictions should be made regarding the prognostic value of a 3MWT for patients following total joint replacement and the time needed to achieve the aforementioned milestones. This study, albeit with a small sample size, provides value for future study design considerations and provides insight into average timelines to achieve functional milestones following a total hip or knee replacement. 

Preoperative measures were not collected in this study, which some studies suggest are important considerations as they impact postoperative outcomes [14]. However, this information may not be available for outpatient physical therapy settings not affiliated with the referring healthcare system. Functional performance test selection should be considered when identifying specified functional milestones with respect to specificity of training. For example, gait speed over a flat surface may not correlate to reciprocally using stairs due to lack of specificity within testing parameters of the selected performance measure and therefore may not be most representative to that milestone. One study shows that those individuals with less than 95 degrees of knee flexion at 12 months post total knee replacement (10.9% of 697 patients) reported significantly decreased levels of function (via WOMAC) compared to those with greater than 95 degrees of motion [15]. The same study showed a weak association between function and knee extension motion at 12 months post total knee replacement with 5% (n=35) of patients demonstrating a knee flexion contracture greater than five degrees [15]. Therefore, we may see limitations or delays in specified functional milestones due to limited range of motion, which a specific functional performance test may not capture. Participant confidence and willingness to perform specified milestones may also not be captured by a functional performance test and could be greatly impacted by preoperative activity levels, functional capacity, or range of motion. Postoperative knee range of motion was one of the most important criteria for patient satisfaction following a total knee replacement and is directly related to postoperative levels of physical activity [16]. Therefore, other clinical measures may need to be accounted for with relation to postoperative functional capacity. 

The authors recognize that multiple factors impact functional ability following a total joint replacement and that exploration of the prognostic value of a single functional performance test, such as gait speed, may not accurately account for all confounding variables. Factors could include differing pain management strategies between patients and institutions, individual pain tolerances, anesthesia type, surgical considerations, comorbidities, prior functional capacity, unique personal factors, and varying socioeconomic determinants are just a few to consider. Education and socioeconomic status have been shown as independent predictors of outcomes in total hip and knee replacement surgeries [1,17]. Preoperative catastrophizing and depression levels are strong predictors for persistent pain following total knee arthroplasty [18]. Collectively, clinicians must consider these variables as they may affect functional milestone achievement. Acknowledgement of these confounding variables is an important consideration into the limitations of this study design. Additionally, this study design explored one functional performance measure, however, there are a multitude of other standardized functional performance tests that could be considered for a similar study design that warrant further exploration.

## Conclusions

The purpose of this study was to explore if gait speed assessed with the 3MWT could provide prognostic insight into the time taken to achieve specified functional milestones following a total hip or knee replacement. A small sample size could have likely contributed to the limited findings in this study. Despite the lack of statistical significance and study limitations, future studies can appreciate study design considerations if assessing prognostic value of standardized physical therapy performance tests to predict timelines to achieve specified milestones following a total joint replacement. Based on the results of this study, inferences should not be made into the predictive value for a 3MWT and the probable time an individual should expect to wean from an assistive device, wean off pain medications, reciprocally use stairs, or walk 30 minutes consecutively. However, average timelines to achieve functional milestones following a total joint replacement were seen within this small sample size and provide valuable information. This data should be interpreted with acknowledgement of study limitations and recognition of confounding variables that may contribute to an individual's functional capacity following a total hip or knee replacement. Further studies are warranted to assess the prognostic value of 3MWT, amongst other performance measures, and expected timelines to achieve specified milestones that patients commonly inquire about following a total joint replacement. 
